# Natural selection versus neutral mutation in the evolution of subterranean life: A false dichotomy?

**DOI:** 10.3389/fevo.2023.1080503

**Published:** 2023-01-25

**Authors:** David C. Culver, Johanna E. Kowalko, Tanja Pipan

**Affiliations:** 1 Department of Environmental Science, American University, Washington, DC, United States; 2 Department of Biological Sciences, Lehigh University, Bethlehem, PA, United States; 3 Karst Research Institute at Research Centre of the Slovenian Academy of Sciences and Arts, Postojna, Slovenia

**Keywords:** natural selection, neutral mutation, troglomorphy, adaptation, *Astyanax*

## Abstract

Throughout the evolutionary tree, there are gains and losses of morphological features, physiological processes, and behavioral patterns. Losses are perhaps nowhere so prominent as for subterranean organisms, which typically show reductions or losses of eyes and pigment. These losses seem easy to explain without recourse to natural selection. Its most modern form is the accumulation of selectively neutral, structurally reducing mutations. Selectionist explanations include direct selection, often involving metabolic efficiency in resource poor subterranean environments, and pleiotropy, where genes affecting eyes and pigment have other effects, such as increasing extra-optic sensory structures. This dichotomy echoes the debate in evolutionary biology in general about the sufficiency of natural selection as an explanation of evolution, e.g., Kimura’s neutral mutation theory. Tests of the two hypotheses have largely been one-sided, with data supporting that one or the other processes is occurring. While these tests have utilized a variety of subterranean organisms, the Mexican cavefish, *Astyanax mexicanus*, which has eyed extant ancestral-like surface fish conspecifics, is easily bred in the lab, and whose whole genome has been sequenced, is the favored experimental organism. However, with few exceptions, tests for selection versus neutral mutations contain limitations or flaws. Notably, these tests are often one sided, testing for the presence of one or the other process. In fact, it is most likely that both processes occur and make a significant contribution to the two most studied traits in cave evolution: eye and pigment reduction. Furthermore, narrow focus on neutral mutation hypothesis versus selection to explain cave-evolved traits often fails, at least in the simplest forms of these hypotheses, to account for aspects that are likely essential for understanding cave evolution: migration or epigenetic effects. Further, epigenetic effects and phenotypic plasticity have been demonstrated to play an important role in cave evolution in recent studies. Phenotypic plasticity does not by itself result in genetic change of course, but plasticity can reveal cryptic genetic variation which then selection can act on. These processes may result in a radical change in our thinking about evolution of subterranean life, especially the speed with which it may occur. Thus, perhaps it is better to ask what role the interaction of genes and environment plays, in addition to natural selection and neutral mutation.

## 1. Introduction

For most biologists, the hallmark of subterranean life is the shared morphology of eye and pigment reduction and loss. As Haldane (1933) pointed out, for every case of progressive evolution, there are likely ten cases of regressive evolution (loss or vestigilization). Despite the universal nature of the process of regressive evolution (Fong et al., 1995), it is most apparent in cave and other subterranean organisms. The long-standing interest in the iconic cave dweller, the European olm *Proteus anguinus*, dating to the 17th century (Shaw, 1999), is primarily due to the absence of eyes and pigment in this salamander. This process of vestigilization and loss of eyes and pigment has occurred in hundreds and probably thousands of lineages, including flatworms, arthropods, mollusks, salamanders, and fish (Culver and Pipan, 2019a). Speleobiologists often use the term troglomorphy (Culver and Pipan, 2019b) to highlight the shared morphology of reduced eyes and pigment (as well as elaborated extra-sensory structures).

The obvious morphological losses of subterranean organisms has led many biologists to suggest non-selective, non-adaptational explanations for these losses. This is not only true for Lamarckians (Lamarck, 1984) and neo-Lamarckians (Packard, 1888) but for Darwinians including Darwin himself (Darwin, 1859), some neo-Darwinians (Poulson, 2017), as well as neutral mutationists (Chakraborty and Nei, 1974; Wilkens and Strecker, 2017). On the face of it, natural selection would seem to be unnecessary when relaxation of selection would seem to be a sufficient explanation. Combined with the apparent relict nature of some subterranean lineages (Humphreys, 2000), the prominence of lost features led some French biologists, especially Jeannel (1943) and Vandel (1964) to propose non-Darwinian, non-selectionist theories of the evolution of subterranean life. Rather than selection, Vandel proposed an internal force (deroulement) leading to the death of phyletic lines, just as individuals die. Neo-Darwinians, beginning in the 1960’s, mounted a vigorous defense of the centrality of natural selection, not only for elaborated extra-optic sensory structures found in many cave-dwelling organisms but also for eyes and pigment (Christiansen, 1961; Poulson, 1963; Barr, 1968). The centrality of natural selection in the evolution of the subterranean fauna was further emphasized by evolutionary developmental biologists, beginning with the pioneering work of Jeffery on the Mexican cavefish *Astyanax mexicanus* (Jeffery, 2009).

*Astyanax mexicanus* has emerged as a central model for examining the mechanisms, both proximate and ultimate, underlying cave-evolved traits such as eye regression and pigment loss. This is due to several reasons, including the presence of a cave and surface form of this species, the amenability of *A. mexicanus* to live and breed in the laboratory, and the rich literature on this species dating back over 70 years (Jeffery, 2020). Much of the early work investigating regressive traits in *A. mexicanus* was performed by Charles M. Breder. While not the discoverer of the Mexican cavefish [that honor belongs to Salvador Coronado (Romero, 2001)], Breder, along with Priscilla Rasquin, played a critical role in promoting *Astyanax* as a model system for the study of eye and pigment loss, especially in the laboratory (e.g., Breder and Rasquin, 1950). In particular, they took advantage of the fact that the cave populations interbred in the laboratory with eyed, pigmented surface river populations. Breder and Rasquin were writing before the emergence of the neo-Darwinian synthesis, spearheaded by Dobzhansky, Simpson, and others in the 1950’s. The question of whether natural selection or genetic drift was the primary driver of eye and pigment loss was not a question in the Kosswig and Kosswig’s (1940). Rather they focused on how cavefish lost eyes, in particular, the physiological mechanisms contributing to eye loss (Breder and Rasquin, 1950). Breder was, however, a keen observer of the ecological conditions under which *Astyanax* cavefish lived firmly held that darkness was the key factor in the evolution of eye regression, which can be the case in either selection or drift models (Breder, 1942; Breder, 1953).

In the last several decades, there has been a heightened interest in the selection/neutrality debate to explain the evolution of regressive cave traits, especially with respect to the work on the Mexican cavefish, made possible not only because it can be bred with its ancestral-like surface counterpart, but also because whole genome sequences are available (McGaugh et al., 2014; Warren et al., 2021) and functional studies can be performed in this species, including manipulation and transplantation of the eye lens in embryos and genetic manipulation (Yamamoto and Jeffery, 2000; Ma et al., 2015; Klaassen et al., 2018). Additionally, there has been the recognition that models of evolution of the eye and pigment in Mexican cavefish and other subterranean species must take into account the likelihood of migration between cave and surface populations in understanding the repeated evolution of cave traits (Herman et al., 2018). Finally, the evidence of the unexpected importance of maternal and epigenetic effects, as well as phenotypic plasticity on morphology (Romero, 2009; Yoshizawa et al., 2012a; Gore et al., 2018; Ma et al., 2018; Bilandžija et al., 2020) calls for an expansion of how the community understands the mechanisms contributing to trait evolution in this species.

The time is propitious for a re-evaluation of this long-standing controversy. We begin with a brief historical review, and then consider epistemological issues surrounding presumed demonstration of the primacy of either selection or neutral mutation. We then consider other ways the previous models were inadequate, and propose a new, broader perspective, one that takes into account how maternal effects and generally how genes and environment interact.

## 2. Historical background

Both Barr (1968) and Romero (2001) review aspects of the history of ideas about the evolution of cave life, but provide very different perspectives and focus on different time periods. Romero extensively reviews the early history, including the neo-Lamarckian school in North America around the turn of the 20th century, as well as the French school of organicism (roughly, the view that evolution is driven by forces above the individual and population level). Barr focuses on 12 theories for eye and pigment loss, most of which had adherents when he was writing in 1968. We focus on the development of what Trontelj (2019) calls the selectionist school of speleobiology, beginning in the mid-1960’s, and subsequent developments.

While the mid-1960’s signaled the birth of molecular population genetics, the first efforts at untangling genetic variation of subterranean organisms at the enzymatic level did not occur until the mid-1970’s (Laing et al., 1976), and reciprocal eye lens transplantation procedures in *Astyanax* were not available until 2000 (Jeffery and Martasian, 1998; Yamamoto and Jeffery, 2002). Thus, the beginnings of the selectionist school relied primarily on the comparative method, with surface and cave species compared with respect to morphology and behavior. The real triumph of Barr, Christiansen, and Poulson was the demonstration that natural selection was a feasible explanation of observed patterns, not that they “proved” selection.

Christiansen (1961, 1965) in fact did not study the eyes and pigment of Collembola. What he did was demonstrate that both morphology and behavior with respect to the ability to walk on water films and surfaces (a presumed advantage in obtaining food) showed convergent evolution in cave species. While this work was before the rise of cladistics (in fact a phylogeny for the Entomobryinae still does not exist), he demonstrated that this convergence (and hence adaptation) occurred in both European and North American lineages. While it may seem strange in a contemporary context, Christiansen gave evidence for adaptation in cave animals, a point disputed by a number of francophone evolutionary biologists (see Vandel, 1964) who held that the cave fauna represented senescent phyletic lines, that were blind not because they were in caves, but were blind and could not survive outside of caves. Christiansen was very much a part of the comparative school, one that Gould and Lewontin (1979) went on to criticize as consisting of “just so” stories of adaptation.

Poulson (1963) employed a similar epistemology in his study of the North American cave fish in the family Amblyopsidae. He looked at a wide range of morphological and behavioral patterns that were present in the cave dwelling species in the family (which includes both surface and cave dwelling species). Rather than look at convergence in different lineages (it was unclear at the time that there was more than one lineage of amblyopsid fish), he used the degree of eye degeneration as a measure of time isolated in caves, and demonstrated correlated levels of adaptation in traits such as lateral line system development, brain structure, and feeding behavior. His use of the degree of degeneration as a measure of time was a common theme among North American neo-Lamarckians, including Eigenmann (1909) who did the original work on eye degeneration of amblyopsids. Poulson placed this all in the context of adaptation to life in darkness with little food. Unlike Christiansen, he does consider eyes, but only as time keepers. Some decades later, Poulson fully adopts a non-selectionist view of eye loss (Poulson, 2017) while retaining his neo-Darwinian credentials.

More than Christiansen or Poulson, Thomas Barr was immersed in the study of cave science (speleology), not only publishing extensively on cave ecology and evolution, but also on species descriptions (as did Christiansen), and the physical description of caves (Barr, 1961). His research was largely focused on systematics and biogeography, but he provided (Barr, 1968) the most comprehensive review of theories of regressive evolution. He lists 12 theories to explain eye and pigment loss, and divides them into three groups: (1) Lamarckian and neo-Lamarckian; (2) Orthogenesis; and (3) Darwinian and neo-Darwinian. Among the eight Darwinian theories, Barr lists three that involve natural selection as the primary motor of change: direct selection, material compensation (energy conservation), and indirect effects of pleiotropy (Krekeler, 1958). Of these, the last two still have currency. Evidence for material compensation and the attendant competition among parts includes changes in relative sizes of brain lobes in amblyopsid fish (Eigenmann, 1909; Poulson, 1963). Evidence for indirect selection due to pleiotropy has come more recently, and includes a number of genes in the visual and pigment pathways of Mexican cavefish, for example, the *oca2* gene in the melanin pathway and the expansion of *shh* expression at the midline, which have both been implicated in contributing to a number of traits (Yamamoto et al., 2009; Bilandžija et al., 2013; O’Gorman et al., 2021). Interestingly, Barr includes two theories involving neutral mutation (genetic drift and accumulation of random mutations) under the Darwinian rubric. His main critique of these mutation drive hypotheses was that there seemed to have been insufficient time for them to lead to trait loss. In some ways, Barr’s summary marks the end of an era. Nearly simultaneously with the publication of his review, Kimura (1968) published a book length exposition on the role of neutral mutation in evolution, Hennig (1965) published the foundation document for cladistics in 1966, and Lewontin and Hubby (1966) ushered in the era of molecular population genetics.

Of course, all of this early work by neo-Darwinists was comparative and correlational. There were no direct tests of hypotheses. The argument for selection had two parts. First, the observed morphological patterns of elaborated characters (such as the claw of Collembola) was elaborated in such a way that was expected in the harsh subterranean environment. Second, losses (eyes and pigment) were unlikely to be the direct result of selection, but energy economy and pleiotropy, both resulting in indirect selection, could explain those losses. Culver et al. (1995) attempted a more rigorous (and falsifiable) test for natural selection in the cave amphipod, *Gammarus minus*, a species that shares with *Astyanax mexicanus* the presence of both surface and cave-dwelling populations that are morphologically distinct. Following Brandon’s (1990) rules for the demonstration of natural selection, they demonstrated heritable variation in eye, antennal, and size traits, and that there was significant selection on all three of these components as measured by fecundity and probability of mating. In a later paper, Christman et al. (1997) demonstrated that the differences in eyes, antennae, and body size could not be explained by phylogenetic effects. While the overall result was at least epistemologically appealing, several aspects of the study remained unexplained. First, there appeared to be selection on eye size but no selective factor is known that would cause this. Second, large antennae, expected to be selected against in surface populations, was selected for.

Finally, the work of Konec et al. (2015) follows in the comparative tradition, albeit with closer attention to phylogeny. They measured 62 morphological traits of the isopod *Asellus aquaticus* for two paired populations from Slovenia and Romania. They found that 18 of 62 traits showed convergence, an indication of the importance of the subterranean environment in molding morphology. Of course these traits included both increases and reductions, and some are classic regressive features such as eye reduction. This study, while fascinating, does not help to distinguish selection from neutral mutation.

While Barr considered mutation theories to be part of neo-Darwinism, its main proponent, Horst Wilkens, clearly saw neutral mutation and genetic drift as an alternative, at least to natural selection as the driving force behind eye and pigment loss. Wilkens’ seminal paper (1971) elaborated and refined ideas proposed by an earlier generation of mutationists, especially Kosswig and Kosswig (1940), who argued, based in part on their studies of the subterranean populations of the isopod *Asellus aquaticus* in Slovenia, that selectively neutral mutations best explained the high levels of variation in eye and pigment.^1^ Relying primary on data on F1 and F2 crosses both between cave and surface, and different cave populations, Wilkens demonstrated polygenic inheritance, independent acquisition of losses, and increased eye variability in darkness. In a career spanning more than 50 years (Wilkens, 1971, 1988; Wilkens and Strecker, 2017), Wilkens has consistently argued that eye and pigment loss does not involve natural selection, but is rather the result of genetic drift and accumulation of selectively neutral, morphologically reducing alleles.

There are several unique features to Wilkens’ view of the evolution of cavefish. First, his argument has remained largely unchanged over a nearly 50 year time span. Based on ideas of the Kosswigs’ about mutation dating back to the 1930’s, the pillar of Wilkens’ arguments is that the increased variability shown by both hybrids and recent cave colonists indicates a relaxation of selection. That is, he equates increased variability with relaxation of selection. Indeed, relaxation of stabilizing selection, in the absence of other complications, will result in an increased variability of the trait, the result of both migration and mutation. However, other kinds of selection will also increase variability, such as disruptive selection. The presumption is that such kinds of selection are rare in the subterranean domain (but see Culver et al., 1994). Second, he pays scant attention to the development of neutral mutation theory by Kimura (1968) even though it provides opportunities for testing hypotheses and refuting the view that there has been insufficient evolutionary time for gene fixation under neutrality. Several researchers have argued that in fact there has been sufficient time for gene fixation under at least some multi-locus models (Chakraborty and Nei, 1974; Culver, 1982; Nei, 2013). Throughout his writing, Wilkens uses degree of morphological degeneration of the eye as a measure of time, rather than testing it directly. Third, he ignores for the most part constructive traits. This emphasis on eye and pigment loss has led some biologists to mistakenly assume that these are the only differences between cave and surface species (see Romero, 2001). It was not until 2001 that a list of constructive as well as regressive traits for *Astyanax* cavefish (Table 1) was published (Jeffery, 2001). An instructive example of how constructive traits were minimized was the statements both by the Schemmel (1967), the original author of the study and by Wilkens, that the increased number of taste buds in cave populations was probably not significant (even though it was statistically significant) since they showed considerable variation.

## 3. The re-emergence of *Astyanax* as a model system and the return of pleiotropy

In the past two decades, a number of advances have been made which allow for experimental manipulation and genetic analysis in *A. mexicanus*. These advances have allowed researchers to further understand the genetic and developmental mechanisms contributing to the evolution of regressive traits in *A. mexicanus* cavefish and to reexamine the role of adaptation in regressive trait evolution in this species.

### 3.1. Studies of adaptation in *Astyanax*

Two mechanisms for how adaptive evolution *via* energy savings could contribute to eye regression in *A. mexicanus* cavefish have been proposed, and both have some support from experimental evidence.

First, it has been proposed that eye regression is adaptive in cavefish because differences in development or maintenance of the eyes provides energy savings (Jeffery, 2005). Cavefish have evolved a number of metabolic adaptations that suggest they have evolved under selective pressures to conserve energy (reviewed in Rohner, 2018). However, few studies have directly addressed whether there is limited food in the caves inhabited by *A. mexicanus* cavefish. Breder, perhaps the most experienced observer of *Astyanax* in caves, doubted that they were food limited (Breder, 1942, 1953), but current conditions in *Astyanax* caves may not reflect conditions at the time of colonization and isolation (Espinasa and Espinasa, 2016). Pipan and Culver (2012) proposed darkness, not food limitation, as the primary selective force. However, in a sense, darkness imposes a food limitation since it makes the finding of food much more difficult, resulting in a virtual scarcity. Further, darkness in caves is universal, whereas food scarcity is not (Pipan and Culver, 2012). As a convenient shorthand, we refer to both these factors as resulting in food scarcity. The energy savings from loss of eyes and pigment could occur through two mechanisms. First, if an organism does not develop eyes, this could result in energy savings during development. In *A. mexicanus* cavefish, eyes are specified and undergo optic cup morphogenesis during early development, albeit with some changes relative to surface fish counterparts, and then degenerate (reviewed in Jeffery, 2005). Further, while the cavefish retina does not increase in size during larval stages like the surface fish retina, this is not due to loss of cell proliferation, but instead due to increased cell death (Strickler et al., 2002). Thus, because the eye is formed and the cells in the retina proliferate, it is unlikely that the evolutionary changes observed in cavefish eye development would provide energetic savings sufficient to drive adaptive evolution of eye loss in this species (Jeffery, 2005), though it should be noted that whether there is energetic savings in cavefish relative to surface fish during development has not been directly tested.

The second mechanism for energy savings due to eye regression is based on the idea that in a nutrient poor environment, there could be a benefit for not maintaining high energy tissues (Niven and Laughlin, 2008). Neural tissues, including those that make up the visual system, are energetically expensive, and thus, reductions in the size of these tissues could be adaptive in a cave environment (Moran et al., 2022). Eyes in adult cavefish are highly degenerate, and fish from multiple populations each have a smaller optic tectum compared to surface fish (Soares et al., 2004; Jaggard et al., 2020). The energetic cost of eyes was measured directly in *A. mexicanus* by Moran et al. (2015). Through direct measurements of metabolic rates of neural tissues, they demonstrated loss of eyes in cavefish would provide substantial energy savings (Moran et al., 2022). Thus, energy savings during visual system maintenance, rather than visual system development, may result in eye loss leading to adaptive benefits in the cave environment.

The genetic mechanisms for the evolution of eye and pigment reduction *via* pleiotropy require a linkage of these reduced traits with adaptive traits. The genetic mechanisms for the evolution of reduced traits *via* energy economy do not necessarily require such linkage. Pleiotropy, where the same gene affects multiple traits, has been the focus of nearly all experimental studies of adaptation in *Astyanax* (see Jeffery, 2005). However, it is important to note that there are other genetic mechanisms that could result in co-variance of traits, i.e., physically linked genes or more generally genes that tend to occur together as a result of linkage disequilibrium. A number of studies are consistent with pleiotropy and indirect selection playing a role in the evolution of regressive traits in cavefish. First, regression of eyes has been linked to expansion of constructive traits that may be beneficial in the cave. The expression of the gene *sonic hedgehog (shh)* is expanded in cavefish relative to surface fish, with a robust expansion at the midline during early development (Yamamoto et al., 2004) and increased expression in neural tissues at later stages of development (Menuet et al., 2007). Manipulation of *shh* signaling during development revealed that *shh* signaling plays a role in eye development and degeneration in this species (Yamamoto et al., 2004). Further, manipulation of *shh* signaling at early developmental stages also affects traits that are enhanced in cavefish, including number of taste buds and jaw size (Yamamoto et al., 2009). They report an observation that is perhaps the most direct evidence for pleiotropy. They found that a surface-dwelling *Astyanax* individual fish contained a heat sensitive expression plasmid with an *shh* gene that produced with eye degeneration or taste bud magnification, depending on temperature. This is the most direct observation of pleiotropy.

Additionally, quantification of jaw size and taste bud number in cave-surface hybrid fish with large or small eyes revealed a relationship between these traits in hybrid fish, further supporting a developmental relationship between these traits that could be mediated by *shh* signaling (Yamamoto et al., 2009). More recently, expanded *shh* signaling has been associated with brain evolution in *A. mexicanus* (Menuet et al., 2007), suggesting this change during development may impact both eye regression and behavior *via* changes to the brain.

In addition to functional studies, quantitative trait loci (QTL) studies suggest that pleiotropy may play a role in the evolution of cave traits, including eye regression. QTL for different cave-evolved traits cluster together within the genome (Protas et al., 2008; O’Quin and McGaugh, 2015). One explanation proposed for these QTL clusters is pleiotropy: that a gene or gene(s) within these genomic locations affects multiple cave-evolved traits (Protas et al., 2008; Yoshizawa et al., 2012b; O’Quin and McGaugh, 2015). One striking example of QTL clustering was identified by Yoshizawa et al. (2012b). Cavefish from some caves have evolved an adaptation associated with feeding, vibration attraction behavior (VAB), the tendency to swim toward a vibrating object in the water (Yoshizawa et al., 2010). The lateral line is necessary for VAB in cavefish (Yoshizawa et al., 2010), and quantification of VAB, the number of lateral line organs, the superficial neuromasts, within the eye orbit (which are found in cavefish and not surface fish), and eye size revealed correlations between these traits in cave-surface hybrid fish, suggesting these traits may share a genetic basis (Yoshizawa et al., 2012b). This was further supported by QTL analysis: QTL for eye size, VAB and eye orbit neuromast number cluster in two locations in the genome, supporting the hypothesis that the same gene(s) contribute to the evolution of all three of these traits (Yoshizawa et al., 2012b). This is significant, as many of the constructive traits that have evolved in cavefish have not been directly linked to a benefit to these fish within a cave habitat. VAB, however, has been shown to provide an advantage when foraging in the dark: Both cavefish and surface fish with VAB struck more at prey in the dark when directly compared to cavefish and surface fish without VAB (Yoshizawa et al., 2010). Thus, VAB may provide an advantage in a cave habitat for foraging, and the selective advantage of alleles that contribute to increasing VAB and neuromast number and reducing eye size could contribute to indirect selection for eye reduction in this species (Yoshizawa et al., 2010, 2012b).

The most definitive evidence for the role of pleiotropy in cavefish evolution comes from studies of the evolution of albinism in cavefish. Multiple populations of cavefish have evolved albinism, defined as the complete loss of melanin pigmentation (Protas et al., 2006). Genetic analysis of albinism suggests that this trait evolved through a single locus of large effect (Șadoğlu, 1957; Protas et al., 2006). The gene responsible for albinism is *oculocutaneous albinism 2 (oca2)*, which was identified initially through QTL mapping, and confirmed through functional studies, including generation of surface fish with mutations in this gene and finding that the resulting animals lacked melanin pigmentation (Protas et al., 2006; Ma et al., 2015; Klaassen et al., 2018). Distinct coding mutations in *oca2* have been identified in two *A. mexicanus* cave populations, and complementation analysis suggests that regulatory mutations in *oca2* cause albinism in a third population (Protas et al., 2006). In addition to playing a role in pigmentation, recent work demonstrates that *oca2* plays a role in behavioral evolution in *A. mexicanus*. Bilandžija et al. (2013) first suggested that mutations in *oca2* may contribute to the evolution of other traits in *A. mexicanus* cavefish. Cavefish have higher levels of catecholamines compared to their surface fish counterparts (Bilandžija et al., 2013; Elipot et al., 2014), and knockdown of *oca2* using morpholinos increases levels of the catecholamine dopamine in larval surface fish (Bilandžija et al., 2013). Catecholamines play a role in regulating a number of behaviors, including sleep and social behaviors (Saper et al., 2005; Scerbina et al., 2012), raising the possibility that *oca2* plays a role in the evolution of these or other cave-evolved behaviors in albino cavefish populations. Indeed, albino fish behave differently from wild-type surface fish. Both depigmented cavefish and hybrid fish selected to be albino and to have eyes have increased levels of catecholamines, and catecholamine-dependent anesthesia resistance compared to surface fish (Bilandžija et al., 2018). Additionally, surface fish engineered to harbor mutations in the *oca2* gene are albino and sleep less than their pigmented, wild-type siblings, suggesting that *oca2* plays a role in both pigmentation and behavior (O’Gorman et al., 2021). While it has been suggested that reductions in sleep are beneficial to cavefish, possibly providing more time for feeding (Duboué et al., 2011), whether reductions in sleep or other behaviors provides a benefit to fish within caves remains to be demonstrated. However, population genetics analysis of the *oca2* locus suggests *oca2* is under positive selection in multiple cavefish populations (O’Gorman et al., 2021). Together, this work provides the most extensive analysis of pleiotropy in this species to date, and suggests that, at least in the case of pigmentation, pleiotropy plays a role in the evolution of cave-evolved traits.

A major caveat to the pleiotropy and indirect selection argument for regressive traits in cavefish is that other genetic factors could explain many of these results: Closely linked genes that independently affect different traits could explain QTL clustering as well as correlations between traits in hybrid fish (Protas et al., 2008; Yoshizawa et al., 2012b; O’Quin and McGaugh, 2015). Even in the case of *shh*, it is not clear that endogenous levels of *shh* in these fish are sufficient to produce these differences in *shh*-dependent traits. Critical to proving indirect selection *via* pleiotropy is to identify and functionally assess the genes and alleles contributing to the evolution of these traits. Identifying the genes near or within QTL remains a significant challenge, particularly for traits that have evolved through multiple loci of small effect size (reviewed in O’Quin and McGaugh, 2015). The sequencing of the *A. mexicanus* cavefish and surface fish genomes has allowed for the identification of a number of candidate genes located within QTL clusters, at least some of which have expression patterns and functional data from other species that is consistent with pleiotropic effects of these genes (McGaugh et al., 2014; Warren et al., 2021). However, functional assessment of these genes and their cave alleles in *A. mexicanus* is critical for understanding if they play a pleiotropic role in cavefish evolution. functional analysis of candidate genes is now possible in *A. mexicanus* using methods that allow for targeted gene manipulation, including TALENs and CRISPR/Cas9 (Ma et al., 2015; Kowalko et al., 2016; Klaassen et al., 2018). However, few candidate genes for regressive traits have been assessed at this level (for exceptions, see Klaassen et al., 2018; Ma et al., 2020; O’Gorman et al., 2021; Warren et al., 2021).

### 3.2. Studies of neutral mutation in *Astyanax*

While the theory of neutral mutation and the related theory of mutation drive evolution is quantitative, and at least in principle, testable, it has been rarely used in actual tests, and rather the general idea of a release of variability following a relaxation of selection has been tested. As we pointed out above, an increase in variability can have different causes.

Chakraborty and Nei (1974) and Nei (2013) estimate, for Pachón Cave in Mexico, whether there has been sufficient time for eye loss to occur. For neutral alleles, the probability of fixation by the *t*-th generation, (P(1,t)), is given by***:

$$P(1,t)=1-(4N\upsilon +1)e^{-\upsilon t},$$

where N is the effective population size and υ the mutation rate per locus per generation. They suggest that υ is of the order 10^−5^ because destructive mutations are selectively neutral, but Nυ is effectively 0 since effective population size is likely to be around 200 (or perhaps larger). If the Pachón cave population diverged about 50,000 years ago and generation time is 5 years, then the probability of fixation of destructive mutations is about 0.63 and υt is approximately 1. Both divergence time and generation time numbers are suspect. Generation time in the lab is approximately 5–6 months. While divergence time is likely much greater than 50,000 years, with the most comprehensive analysis to date suggesting between 161,000 and 191,000 generations divergence between cave and surface populations (Herman et al., 2018), one study suggests that divergence may be as little as 20,000 years (Fumey et al., 2018). Reduced generation time makes the probability of fixation more likely while shortened divergence times do the opposite. This, and other similar tests for multiple loci (Culver, 1982), show a concordance with neutral theory, not necessarily a test of its validity.

Using Lande’s (1976) work on the rates of phenotypic evolution under genetic drift, Culver (1982) used Wilkens’ (1971) data on eye diameters to estimate that effective population sizes could be no larger than 270 *h*^2^ individuals (where *h*^2^ is heritability) for drift to account for the observed changes. If heritability is close to 1, then effective population sizes are in the range of estimates for Pachón Cave, but the mean estimate is about twice as large (Bradic et al., 2012). As Culver (1982) pointed out, there really is not sufficient information for a more rigorous test, and this has not changed in the intervening 40 years.

Certainly the central figure on the neutral evolution side of the debate about the evolution of cave populations of *Astyanax* is the German geneticist, Horst Wilkens, arguing forcefully for the importance of neutral processes for nearly 50 years. In the recent book length treatment of *Astyanax* (Wilkens and Strecker, 2017), he departs from his usual position of pointing out the areas of agreement with neutral theory and includes a critique of selectionist ideas, especially those involving pleiotropy. Theirs is a like a meta-analysis, with arguments taken from a series of papers on pleiotropy. This critique stands alone and they accept precious little of current work on pleiotropy, and it is difficult to judge this objectively. They do raise important points, especially the need to demonstrate pleiotropy with the association of QTLs for constructive traits with major genes of eye development such as *shh*.

### 3.3. Studies of natural selection and neutral mutation

Cartwright et al. (2017) put forward a general model of the evolution of eye (or pigment) loss in *Astyanax* in which gene frequencies are affected by directional selection (either energy economy or pleiotropy), as well a migration of large-eyed surface dwelling fish. The inclusion of migration is important for *Astyanax* because surface and cave populations are interfertile and in close proximity. This is not universally true for cave-limited species but in the model, migration can be set to zero. Their basic model is as follows:

$$q_{j}=\{(1+s)q^{2}+(1+hs)q(1-q)\}∕\{(1+s)q^{2}+2(1+hs)q(1-q)+(1-q{)}^{2}\}$$

selection

$q_{a}=q_{j}(1-m)+Qm$ immigration

$q^{\prime }=q_{a}+(1-q_{a})u$ mutation

$q_{j}=$ the frequency of the allele that causes blindness as a result of selection; $q_{a}=$ the frequency of the allele that causes blindness as a result of migration; $q^{\prime }=$ the frequency of an allele that causes blindness in the cave population in the next generation due to selection, migration and mutation; q= allele frequency of an allele that causes blindness; s= fitness advantage of an allele that causes blindness; h= dominance level of an allele that causes blindness; m= the rate of immigration from the surface population to the cave populations; Q= the allele frequency of an allele that causes blindness in the surface population; u= the mutation rate of an allele that causes sightedness to on that causes blindness.

Even without the complication of multiple alleles and dominance, they show that there can be equilibria at 0, 1, or multiple interior points, depending on the intensity of selection. Among their general conclusions are that immigration rates must be less than mutation rates for neutral processes to predominate; that s must be greater than 48 times the immigration rates for selection to predominate, and that if the population is younger than 1∕υ (the inverse of the mutation rate), selection rather than neutral mutation will predominate. In a test with real data, Herman et al. (2018) show that selection coefficients must be 0.01 to drive frequencies of blind alleles toward fixation. These are at least feasible values. Of course, different parameters from the ones Herman et al., used may yield different results. In particular, they posit a recent origin for population splits— approximately 175,000 generations.

Borowsky (2015) claimed that a test based on one developed by Orr (1998) could distinguish between selection and neutral mutation by examining the polarity QTL. In particular, the claim that nearly all QTL had negative polarity (cave alleles were structurally reducing) for eye size and mixed polarity for melanophore number indicates selection was responsible for eye size reduction but neutral mutation was responsible for melanophore reduction, is not necessarily indicative of different processes, but rather the nature of mutations arising in the populations (Culver, 1982). Lande (p. 194 in Wilkens and Strecker, 2017) provides a more general critique of Orr’s test, as does (Poulson 2017).

## 4. Emerging themes

A number of themes emerge from the often fractious literature on the evolution of *Astyanax* in caves.

First, some biological features of *Astyanax* make it an ideal model system for the study of gains and losses in evolution, not just in caves, but in general. The reduction and/or loss of eyes and pigment are among the most obvious examples of losses (regressive evolution) in the animal kingdom. Likewise, the harsh environment of caves, especially the absence of light (which makes food scarce in caves in terms of the ability of organisms to locate it) points to the direction of constructive evolution.

Second, *Astyanax* is one the few cave species that is interfertile with its surface ancestor [the isopod *Asellus aquaticus* is another (Protas and Jeffery, 2012)] allowing for a range of genetic experiments unavailable in other species. In spite of concerns that this makes it atypical (Poulson, 2010), it also makes much work possible.

Third, the kind of neutral evolution modeled by Kimura, and to a lesser extent by Nei, is inevitable. Similarly, directional selection is almost inevitable as well, as long as there is differential fitness of alleles, a fact little disputed for constructive traits, and increasingly demonstrated for features of eye and pigment reduction. The question is whether there has been sufficient time and sufficiently strong selection for either of these evolutionary mechanisms to be quantitatively important.

Fourth, with few exceptions (e.g., Cartwright et al., 2017), there have not been tests of whether either neutral mutation or selection has occurred, but rather whether they are feasible. In particular, models of selection or neutral mutation can, but not inevitably, yield reasonable values of time required.

Fifth, the breadth of the study often determines the outcome. For example, the Hamburg research group headed by Wilkens produced no list of adaptive features—that did not occur until Jeffery did so in 2001. Even when adaptive features were studied by the Wilkens group, their impact was minimized (e.g., Schemmel, 1967). Likewise, studies of pleiotropy ignore neutral mutation, and the very design of experiments on both sides often precludes their joint consideration.

Sixth, there are now a number of studies that demonstrate or at least show the feasibility of both selection and neutral mutation in eye and pigment systems. While interesting, more demonstrations of either process likely will not expand our understanding of the entire evolutionary process.

Seventh, there are processes, especially epigenetic effects, environmental effects, and repeated migration, that have been mostly ignored, yet have proven important (Gore et al., 2018; Herman et al., 2018; Bilandžija et al., 2020). Perhaps the most transformative of these is the potential for *hsp90* to facilitate colonization of caves (Rohner et al., 2013).

## 5. A new path forward

*Astyanax* is certainly the most thoroughly studied cave organism in the world. A Pubmed search (accessed 25 October 2022) of “Astyanax” lists 753 publications. However, our knowledge of the species *Astyanax mexicanus* is highly uneven. While there are a large number of papers on genetics and development, there are relatively few papers on ecology, especially differences in environment among caves and population genetics, particularly in a molecular genetic context. We find several areas that are in need of attention and hold promise for new discoveries, outlined below.

First, while a number of loci have been identified for cave-evolved traits in *A. mexicanus* (reviewed in O’Quin and McGaugh, 2015), relatively few causative genes and genetic variants that contribute to these traits have been identified (for exceptions, see: Protas et al., 2006; Gross et al., 2009; Klaassen et al., 2018; Ma et al., 2020). Identification and functional testing of genes contributing to cave-evolved traits is critical for understanding how evolution has shaped cave *A. mexicanus*, and phenotypic analysis of animals harboring mutations in these candidate genes has the potential to illuminate their role in underpinning these traits, as well as whether these genes and alleles have pleiotropic effects (for example, see O’Gorman et al., 2021). Both cave and surface *A. mexicanus* now have published genomes (McGaugh et al., 2014; Warren et al., 2021), and the demonstration that tools for functional genetic analysis, including TALENs, CRISPR-Cas9, and transgenesis, work in this species (Elipot et al., 2014; Ma et al., 2015; Klaassen et al., 2018; Stahl et al., 2019), provide a path forward for this type of analysis.

Except for very early work shortly after the discovery of the species (especially Breder, 1942), little comment has been made about the cave environments where *A. mexicanus* live. Eliott (2015, 2018) provide a detailed description of *A. mexicanus* caves, but this is mostly limited to maps and their interpretation. Most authors have simply assumed that the caves are food poor and without light (Jeffery, 2020). However, Breder (1942, 1953) argues against food limitation, at least in la Cueva Chica, where he did extensive preliminary studies. Few studies have addressed food availability in the caves directly (for exceptions, see Espinasa et al., 2017; Wilson et al., 2021). Even less studied are differences among the caves, which may explain some of the variation seen. There have been hints of interesting environmental variation between localities [e.g., temperature and oxygen (Rohner et al., 2013; Ornelas-García et al., 2018; Tabin et al., 2018; Krishnan et al., 2020)]. Particularly interesting is the suggestion by Rohner et al. (2013) that reduced conductivity in cave waters acts as a trigger to express variation hidden by norm of reaction. Further, differences between microhabitats within caves, such as between different pools within caves, could have ecological and evolutionary significance (for example, Trontelj et al., 2012; Borko et al., 2021).

Understanding the ecological conditions of the cave can provide additional insight into how these environmental conditions affect phenotypic traits. The pioneering work of Jeffery and his colleagues (Gore et al., 2018; Bilandžija et al., 2020) demonstrated the importance of phenotypic plasticity and epigenetic effects in the evolution of cave traits. Somewhat earlier, Romero, 2009 argued that phenotypic plasticity is the key to understanding the colonization of caves in general. Additional studies elucidating the mechanisms underlying this plasticity, as well as response to other environmental factors found in cave habitats, will further our understanding of the role of these processes in the evolution of cave traits.

With a few notable exceptions (Chakraborty and Nei, 1974; Nei, 2013; Borowsky, 2015; Cartwright et al., 2017; Herman et al., 2018), mathematical population genetic models have not been used in the study of evolution of *Astyanax*. These models generally provide tests of the feasibility of particular evolutionary processes in explaining the evolution of morphology and genetics in *Astyanax* populations. Whole genome sequencing of wild-caught fish genomes (as in Herman et al., 2018), is now feasible, and will provide additional information about how cavefish traits have evolved. Further, analysis of *Astyanax* genomes has the potential to provide insight into genome evolution, such as the role of structural variation in the evolution of cavefish (for example, see Warren et al., 2021).

The study of cave life has been plagued by an ever increasing jargon used to describe many aspects of the evolution of cave animals, including convergence, specialization, and distribution (Culver et al., 2023). For example, the phrase “troglomorphic” used to describe characters correlated with subterranean life can easily be replaced by the more general evolutionary term convergence. The effect of this jargon is to diminish the true generality of the results, and appears to directly affect the readership (Martínez and Mammola, 2020). While there has been recent interest in the generality of the *Astyanax* model with respect to human disease related traits (Rohner, 2018; McGaugh et al., 2020), its generality with respect to evolution in extreme environments, such as caves, as not been stressed. This we believe is unfortunate because *Astyanax* holds considerable promise as an exemplar for evolution in extreme environments as well as for understanding basic evolutionary processes.

## 6. Is *Astyanax* an appropriate model of evolution of cave life?

Ever since its discovery more than 80 years ago, *Astyanax mexicanus* has had a rather ambiguous place in field of speleobiology. It is after all not an iconic cave organism, in the sense that the European cave salamander *Proteus anguinus* is. It does not share with *Proteus* and many other cave dwellers the other-worldly appearance and extremity of specialization. *Proteus* is almost the antithesis of *Astyanax; Proteus* has no known surface ancestors, reaches sexual maturity after decades, and is usually placed in a separate family, the Proteidae. Some of the very features that make *Astyanax* such a useful model system make it unique and therefore suspect. These include its ready hybridization with surface populations (actually not a unique feature) and the possibility of genetic and developmental manipulations. The separation of *Astyanax* research from other speleobiological research is exacerbated by the fact that work on *Astyanax* is laboratory intensive and much other work is field intensive.

The standard view is that *Astyanax* represents an earlier stage of adaptation of subterranean life than the extreme specialists like *Proteus* and the North American cave fish family Amblyopsidae, and that the processes involved (e.g., natural selection) are the same. Indeed, the isopod *Asellus aquaticus* shows many of the pattern of *Astyanax* in caves (Protas and Jeffery, 2012). However, it is possible that old species like *Proteus anguinus* have evolved by different pathways. Perhaps the continued presence of surface ancestors makes for a fundamental difference, or it may be that tropical and temperate cave populations face different challenges and hence different solutions. We think that this is unlikely, but while it is clear that all of the answers about the evolution of cave life cannot be answered with *Astyanax*, we believe many of them can. What phylogenetically old species can add in particular are answers to the following:

1. Does most morphological change occur rapidly followed by a long period of relative stasis?
2. What is the pattern of post-colonization dispersal?
3. Is there evidence of a fundamentally different path to adaptation to subterranean life than that of *Astyanax?*

If *Astyanax* is an appropriate model system, it has huge advantages (Jeffery, 2019)—crossing with surface-dwelling individuals, developmental and genetic accessibility, and the availability of whole genome DNA sequences. However, work on old subterranean groups yields unique insights as well. Only through studying additional cave lineages and other organisms evolving in extreme environments will we understand how generalizable (or unique) what we have learned about the genetic and evolutionary mechanisms that drive evolution of cave populations of *A. mexicanus* are.

## Figures and Tables

**TABLE 1:** Constructive and regressive changes in *Astyanax* cavefish, modified and simplified from Jeffery (2001).

Trait	Type of change
Cranial neuromasts	Constructive
Egg size	Constructive
Fat content	Constructive
Infraorbital bones	Constructive
Larval jaw	Constructive
Maxillary teeth	Constructive
Taste buds	Constructive
Telencephalon	Constructive
Aggressive behavior	Regressive
Circadian activity	Regressive
Eyes	Regressive
Metabolism	Regressive
Optic tectum	Regressive
Pigmentation	Regressive
Pineal gland	Regressive
Schooling behavior	Regressive
Scales	Regressive
Vertebrae	Regressive

## Footnotes

1 The contemporary interpretation of the populations the Kosswigs studied indicates a complex pattern resulting from at least three colonizations of the Postojna Planina Cave System (Slovenia) by *Asellus aquaticus* (Verovnik and Konec, 2019).
